# Physical activity enjoyment as a mediator between weight status and physical activity in children and adolescents: results of the MoMo 2.0-Study

**DOI:** 10.1186/s12887-026-07090-0

**Published:** 2026-06-04

**Authors:** Lara Tschuschke, Carmen Volk, Darko Jekauc, Anke Hanssen-Doose, Claudia Niessner, Alexander Woll, Janis Fiedler

**Affiliations:** 1 Institute of Sports and Sports Science, Karlsruhe Institute of Technology, Engler-Bunte-Ring 15, Karlsruhe, 76131 Germany; 2 Institute of Movement and Sport, Karlsruhe University of Education, Bismarckstr. 10, Karlsruhe, 76133 Germany

**Keywords:** Children, Physical activity, Physical activity enjoyment, Body mass index, Overweight, Underweight

## Abstract

**Background:**

Children with underweight or overweight show different physical activity (PA) levels than their peers with normal weight, and PA enjoyment may explain this difference. This cross-sectional study examined whether PA enjoyment mediates the association between weight status and PA in children and adolescents who are overweight or underweight compared to those with normal weight.

**Methods:**

We analysed questionnaire-based data from 3,718 participants (52.4% male, mean age 10.4 ± 3.7 years) and accelerometer-based data from 1,531 participants (52.1% male, mean age 10.7 ± 3.6 years) of the nationwide MoMo 2.0-Study in Germany (2023–2024). Weight status was categorized according to cut-offs of the International Obesity Task Force into underweight, normal weight, and overweight. PA enjoyment was measured with the short version of the Physical Activity Enjoyment Scale, PA was assessed via MoMo-Physical Activity Questionnaire and with accelerometers (activPAL4). Mediation analyses were performed using Hayes PROCESS mediation macro for total, direct, and indirect effects, adjusted for age, sex, and socioeconomic status.

**Results:**

PA enjoyment fully mediated the relationship between overweight and PA in questionnaire-based (indirect effect = − 0.104, 95% CI [–0.135, − 0.075]) and accelerometer-based data (indirect effect = − 0.065, 95% CI [–0.100, − 0.034]), compared to children with normal weight. For underweight, a small indirect effect emerged for questionnaire-based (–0.035, 95% CI [–0.068, − 0.003]), but not for accelerometer-based data (–0.025, 95% CI [–0.055, 0.003]).

**Conclusions:**

These findings suggest that reduced PA enjoyment partly contributes to lower PA levels among children with overweight, emphasizing the role of motivational factors in this pathway. For underweight, findings were less consistent. Interventions to promote PA in children with overweight may benefit from targeting PA enjoyment as a key motivational pathway.

## Background

The prevalence of unhealthy weight status among children is notable: approximately 25% of children in Europe have overweight or obesity [1] and around 8–9% have underweight [2]. Children with overweight show higher rates of mental disorders and lower self-esteem, compared to children with normal weight [3]. The same applies to adulthood, where a high Body Mass Index (BMI) is a leading risk factor for disability-adjusted life years and deaths in high-income countries [4]. Conversely, underweight during childhood may pose health risks for growth and development, including heightened susceptibility to infectious diseases [5]. In girls and women, low body weight is linked to menstrual irregularities, raising risks of infertility and miscarriage [6]. Both conditions, underweight and overweight, increase health care costs.

A key aspect in understanding the consequences of weight status in childhood lies in its association with physical activity (PA). It is well known that children with overweight engage in less moderate to vigorous PA (MVPA) than children with normal weight, limiting their opportunity to benefit from the health-promoting effects of PA [7]. These patterns can be attributed to various barriers to PA in children with overweight, including social constraints (e.g. lack of support, stigmatization, chaotic home environments), individual obstacles (negative body image, fatigue, low confidence), and motivational issues (unrealistic goals, low self-efficacy) [8]. For children with underweight, mixed results emerge: Some studies report lower levels of PA regarding organized PA, but not for unorganized PA [9], while others found differences only in boys [10] or in girls [11] when comparing PA with children of normal weight.

Another possible explanation for lower PA in individuals with overweight and obesity could be that excess weight makes PA less enjoyable than in individuals with normal weight [12]. PA enjoyment is associated with PA behaviour in children [13, 14] and is described as a positive emotional experience, characterized by feelings such as pleasure [15]. This construct is also taken into account in theoretical models to explain PA behaviour in youth, like the Weiss-Harter model [16]. Adapted from Harter’s self-esteem framework, the Weiss-Harter model links perceived competence and social support to self-esteem, which in turn influences PA enjoyment and PA behaviour. PA enjoyment is highlighted as a key driver of long-term PA involvement [16]. Jekauc and colleagues demonstrated a positive association of PA enjoyment with PA within the Weiss-Harter model in children [17]. Children with overweight and obesity have often been found to report lower levels of PA enjoyment [18], and recent evidence suggests that higher BMI is linked to lower PA enjoyment across different age groups [19]. In contrast, one study reported higher PA enjoyment among children with underweight compared to those with normal weight [20]; however, this finding should be interpreted with caution given the small sample size, the specific Malaysian cohort, and limited methodological detail, which restrict comparability with other populations. Yet, other studies found no differences between children with and without overweight [21], suggesting that the role of PA enjoyment may be more complex. These mixed findings indicate that PA enjoyment may not simply differ by weight status, but could act as a motivational factor influencing the extent of PA in children and adolescents.

Taken together, these findings suggest a potentially mediating role of PA enjoyment in the association between weight status and PA. Mediation refers to an indirect pathway, where weight status affects PA not directly, but through its influence on how enjoyable PA feels. While several studies have examined the individual associations between these constructs, research exploring their combined and interacting effects is still scarce. A cohort study from Italy showed that physical self-perception mediated the association between BMI and PA in children. However, whether PA enjoyment serves as a mediator in children and adolescents with overweight or obesity has not yet been clarified [22]. There remains a research gap regarding positive emotional responses to PA in children with different weight statuses, especially concerning those with overweight or obesity [23] and underweight [20].

**Fig. 1:**
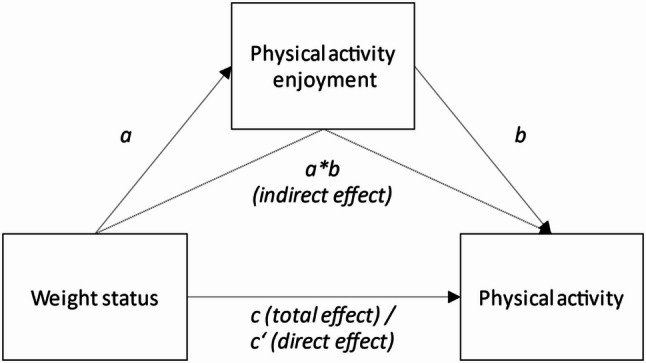
Path diagram for the simple mediation model (theoretical model)

Understanding the role of PA enjoyment may help tailor interventions that not only promote PA but also make it more appealing and improve the sustainability of intervention effects, especially for children with overweight or obesity. To address this gap, we investigated the potential mediating role of PA enjoyment in the association between weight status and PA among children and adolescents aged 4 to 17 years, using data from the nationwide MoMo 2.0-Study in Germany (2023–2024). We hypothesized that the difference in PA (Y) among children and adolescents with overweight and obesity (D_1_) or underweight (D_2_) compared to children with normal weight was mediated by PA enjoyment (M) (Fig. 1). Furthermore, we differentiated between self-reported and device-based PA.

## Methods

### Study design

The MoMo 2.0-Study is a nationwide cohort study collecting representative data on PA, motor performance, and health outcomes in children and adolescents in Germany [24]. It combines survey-based assessments with device-based PA measurements using accelerometers. A two-stage stratified random sampling procedure was implemented with GESIS (Leibniz Institute for the Social Sciences). First, 185 sample points across 171 municipalities were selected. The 185 sample points were, in a first step, allocated proportionally to the 3–17-year-old population in each federal state, receiving stratum weights which were rounded to integers via the Cox algorithm [25]. Within each stratum, municipalities were chosen with a probability proportional to the number of children in the target age group. In the second stage, local registration offices provided contact details of 10 randomly selected children in each of the 14 age groups of 4–17-year-olds, resulting in 140 children per sample point. Children were invited by mail, and data were collected between September 2023 and December 2024. Participants provided written consent (for those under 16 by a parent or legal guardian). They completed the MoMo Physical Activity Questionnaire (MoMo-PAQ) [26] and underwent anthropometric and motor performance assessments conducted by trained staff. Afterwards, participants were asked to wear an accelerometer for 8 days. Trained staff conducted a face-to-face health interview with the participants with questions regarding physical and mental health and socioeconomic status. Participants were tested on-site. If on-site testing was not possible due to illness or similar, a shortened online version of MoMo-PAQ and the health interview was provided. Of 27,865 invited children, 4,405 participated (15.8%). Detailed information is available in the study protocol [24].

### Ethics and dissemination

The study has been performed according to the Declaration of Helsinki. Ethical approval for MoMo 2.0-Study was obtained from the Ethics Committee of the Karlsruhe Institute of Technology on November 6, 2023 (Approval No. A2023-077).

### Registration details

The MoMo 2.0-Study was registered in the NFDI4health database (NFDI4health, 2023; https://csh.nfdi4health.de/resource/1034).

### Preregistration

The study was preregistered on the Open Science Framework (OSF; registration DOI: 10.17605/OSF.IO/VN24H; https://osf.io/vn24h).

### Participants

Children who were unable to participate in the motor performance tests due to health conditions were excluded from data collection. Participants and their parents or legal guardians needed adequate German language skills to follow study procedures. Participants between 4 and 17 years, who were tested on-site and had no missing values on any of the variables used in the analysis, were included. Participants who specified neither male nor female as their biological sex were excluded from our analysis, because it is not yet possible to accurately classify the weight status.

### Measures

#### Body mass index (predictor D_1_ and D_2_)

Anthropometric measurement followed a standardized protocol [27]. Trained staff measured body weight using a digital scale (Seca 813, in kg, to the nearest 0.1 kg) (Seca, Hamburg, Germany) and body height using a stadiometer (Seca 213, in cm, to the nearest 0.1 cm) (Seca, Hamburg, Germany). Participants were measured in light clothing without shoes. The highest and lowest 10% of weight and height values were checked for logical errors and plausibility. BMI was calculated (kg/m²) and weight status was categorized into overweight, normal weight, and underweight according to the age- and sex-adjusted cut-offs of the International Obesity Task Force (IOTF) [28].

#### Physical activity enjoyment (mediator variable M)

PA enjoyment was assessed using the short version of the Physical Activity Enjoyment Scale (PACES-S) [15]. In previous research, positive correlation with both questionnaire-based (MoMo-PAQ) and accelerometer-based measures of PA was reported, as well as a good test-retest reliability and internal consistency [15]. The scale consists of four items: “I enjoy it”, “I find it pleasurable”, “It is very pleasant”, and “It feels good”. Responses were given on a 5-point Likert scale: 1 = “strongly disagree” to 5 = “strongly agree”. The mean value for each participant was calculated based on the four answers to the Likert scale.

#### Physical activity (dependent variable Y)

To measure PA via questionnaire, participants were asked how many days of a normal week they obtained MVPA for at least 60 min per day (excluding curricular sports) [26, 29]. To explain what constitutes MVPA, participants were given the following definition and examples in the questionnaire: “Physical activity includes any activity that causes the heart rate to increase and breathing to increase for a period of time. PA can include sports, playing games with friends, or walking to school daily. Some examples include: running, strenuous hiking, roller skating, cycling, dancing, skateboarding, swimming, playing basketball, soccer, and surfing.” Responses were given on an 8-point scale: 1 = “0 days” to 8 = “7 days”. A previous study reported moderate test-retest reliability and moderate correlation with accelerometer-based MVPA [30].

Furthermore, we included accelerometer-based data to account for PA. Participants were instructed to wear a thigh-mounted accelerometer (activPAL4, PAL Technologies Ltd., Glasgow, Scotland) continuously for 8 consecutive days, including during sleep and water-based activities such as showering or swimming. The activPal4 assesses the 24-hour physical behaviour [24]. The lightweight triaxial accelerometer captures raw acceleration data within a range of ±4 g at a sampling frequency of 40 Hz. Exclusion criteria for wearing an accelerometer were a plaster allergy, skin irritation, as well as an upcoming MRI or a security check (e.g., at the airport). To ensure waterproofing and adherence, the device was sealed in the finger of a disposable glove and affixed directly to the skin using a transparent adhesive dressing (Tegaderm, 10 × 8 cm). The sensor was placed on the front of the right thigh, 10 cm above the patella, with the leg bent at 90°. Trained staff placed the accelerometer on the participant’s thigh to ensure consistent and accurate positioning. Data were recorded continuously without epoch segmentation and stored in onboard memory (64 MB). Data were downloaded and processed using the PAL Software Suite. PALconnect was used to download data, PALbatch was used to export the raw data as .csv. From the recorded raw 24-hour data, we used ActiPASS [31], a validated and freely accessible software [32] designed for processing data from thigh-worn accelerometers, incorporating methods for detecting non-wear time and sleep [33]. We added an age-specific adaptation of the cadence threshold for MVPA [34], and derived a variable estimating the average daily time spent in MVPA across the wear period. Data quality was visually checked for all records flagged according to the ActiPASS criteria. We excluded cases with implausible data (e.g., due to an incorrect reference position). If such issues occurred only on specific days, only data from those days onwards was excluded. The processed daily output was then aggregated in R [35] and RStudio [36] to derive weekly average values per day. A valid day was defined as at least 20 h of recording time. To be included in the analyses, participants were required to have a minimum of two valid weekdays and one valid weekend day, representing a valid measurement week [37]. Further details about using accelerometer data in MoMo 2.0 can be found elsewhere [38].

#### Covariates

Variables for age (in years), sex, and socioeconomic status (SES) were used as covariates. Age influences the amount of PA in childhood [39]. Further, boys and children with higher SES generally report higher PA than girls and children with lower SES [40, 41]. The children were asked about their biological sex via MoMo-PAQ. SES was derived according to Lampert and colleagues by assigning scores from 1 to 7 to the highest educational attainment (combination of school and vocational education), the higher current occupational status of the two parents (combination of position and supervisory responsibility), and the equivalized net household income, which was asked during the health interview. The three component scores were summed to obtain the SES score (range 3–21; complete case approach). SES was then categorized based on quintiles: the lowest 20% were classified as low SES, the middle 60% as middle SES, and the highest 20% as high SES [42].

### Statistical analysis

We used R (version 4.4.2) and RStudio (version 5.1.513) [35, 36] to prepare and conduct our analysis. We calculated BMI percentiles according to IOTF by using the package childsds in RStudio [36]. Then, we categorized weight status by using the specific cut-offs according to IOTF. We generated absolute frequencies (n) and relative frequencies in percent (%) for describing the study population and to present the percentage of weight status categories according to IOTF for categorical variables. Means and standard deviations (SD) were calculated for continuous variables. To ensure sufficient case numbers, the IOTF categories thinness grade 1, thinness grade 2, and thinness grade 3 were merged into one underweight group, and overweight, obesity, and severe obesity into one overweight group, yielding three categories: underweight, normal weight, and overweight.

Mediation analysis with two models (one with questionnaire-based data, one with accelerometer-based data) was conducted to examine whether the association between weight status and PA is dependent on PA enjoyment. For estimating the simple mediation model via linear regression, we used the Hayes PROCESS macro (version 4.3.1) for mediation (model 4) in R [43]. For both models, we used the three categories of weight status as a predictor. Normal weight was used as the reference group, and the two remaining categories were dummy-coded variables (D_1_ for overweight and D_2_ for underweight). We examined total effects of weight status on PA (*c*_*1*_, *c*_*2*_) as well as direct effects of weight status on PA enjoyment (*b*) and direct effects of PA enjoyment on PA (*a*_*1*_, *a*_*2*_). Then, we tested for direct effects of weight status on PA while controlling for PA enjoyment (*c*′_*1*_, *c*′_*2*_) and indirect effects for overweight (*a*_*1*_ × *b*) and underweight (*a*_*2*_ × *b*). Both models were adjusted for all co-variables (age, sex, and SES). For indirect effects, 10,000 bootstrap samples were used. We interpreted the mediation as present if the 95% bootstrapped confidence interval (CI) did not include zero. We reported unstandardized and standardized regression coefficients for paths *a*_*1*_, *a*_*2*_, *b*, *c*, *c*′_*1*,_ and *c*′_*2*_. For indirect effects, we reported unstandardized and – due to dichotomous predictors – partially standardized relative indirect effects with 95% CI (*a*_*1*_ × *b; a*_*2*_ × *b*). Data analysis was based on a complete-case sample (listwise deletion), excluding participants with missing values on any of the variables used in the analysis. For the calculation of the mean score of the PACES-S, listwise deletion was applied, meaning that only participants with complete data on all four items were included in the analysis. Given that the association between age and PA may not be linear across childhood and adolescence, we explored modelling age as a second-order polynomial (i.e., including linear and quadratic age terms) in all regression models as sensitivity analysis. Based on R² and standardized coefficients, results did not differ between these two models, and only the model with a linear trend for age is reported.

## Results

**Fig. 2:**
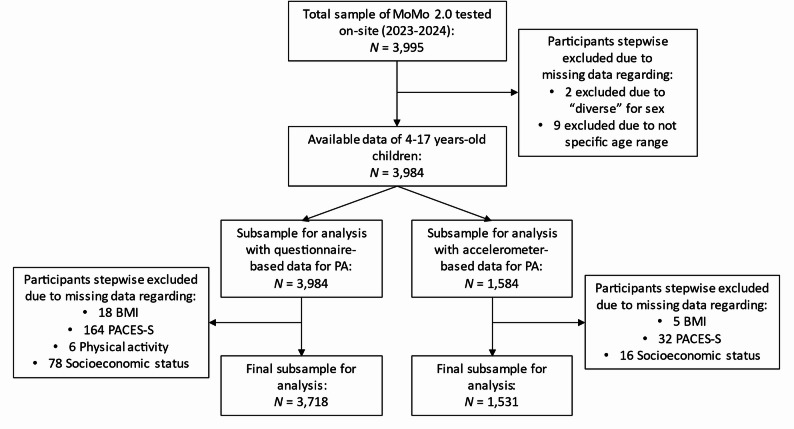
Flowchart of stepwise exclusion for the study sample

Details of the participant selection process for the following analysis, including stepwise exclusions, are represented in Fig. 2. Table 1 shows the sociodemographic characteristics of the study population. Of the original 4,405 study participants, a total of 3,718 participants were included in the analysis with questionnaire-based data (52.4% male, mean age 10.4 ± 3.7 years). As expected, due to the process of creating SES categories, participants were almost evenly distributed in terms of SES in 19.5% low, 60.7% middle, and 19.8% high SES. According to the definition of IOTF, 9.0% of the participants were underweight and 16.1% overweight. Regarding accelerometer-based data, 1,531 children fulfilled eligibility criteria, the ActiPASS criteria for plausible data and the valid week criteria and were included in the analysis (52.1% male, mean age 10.7 ± 3.6 years).

**Table 1:** Sociodemographic characteristics in 4–17-year-old children in Germany

	Total				Girls				Boys			
	Questionnaire-based data (*N* = 3718)		Accelerometer-based data (*N* = 1531)		Questionnaire-based data (*n* = 1770)		Accelerometer-based data (*n* = 789)		Questionnaire-based data (*n* = 1948)		Accelerometer-based data (*n* = 733)	
Age, mean (± SD)	10.4	(3.7)	10.7	(3.6)	10.4	(3.8)	10.8	(3.7)	10.4	(3.7)	10.6	(3.5)
Age category, N (%)												
4-5 y	539	(14.5)	162	(10.6)	260	(14.7)	86	(10.8)	279	(14.3)	76	(10.4)
6-10 y	1576	(42.4)	684	(44.7)	757	(42.8)	353	(44.2)	819	(42.0)	331	(45.2)
11-13 y	860	(23.1)	361	(23.6)	398	(22.5)	178	(22.3)	462	(23.7)	183	(25.0)
14-17 y	743	(20.0)	324	(21.1)	355	(20.1)	181	(22.7)	388	(19.9)	143	(19.5)
Socioeconomic status, N (%)												
Low	726	(19.5)	257	(16.8)	341	(19.3)	134	(16.8)	385	(19.8)	123	(16.8)
Middle	2255	(60.7)	937	(61.2)	1076	(60.8)	487	(61.0)	1179	(60.5)	450	(61.4)
High	737	(19.8)	337	(22.0)	353	(19.9)	177	(22.2)	384	(19.7)	160	(21.8)
Anthropometrics, mean (± SD)												
Height (cm)	143.1	(21.7)	144.8	(20.7)	141.6	(20.4)	144.0	(19.5)	144.5	(22.8)	145.7	(21.9)
Weight (kg)	39.3	(17.6)	40.0	(16.8)	38.4	(16.6)	39.7	(16.0)	40.2	(18.5)	40.3	(17.7)
BMI (kg/m²)	18.1	(3.5)	18.1	(3.4)	18.1	(3.6)	18.3	(3.5)	18.1	(3.5)	18.0	(3.2)
Weight status category (IOTF), N (%)												
Thinness (grade 1 to 3)	334	(9.0)	146	(9.5)	180	(10.2)	87	(10.9)	154	(7.9)	59	(8.0)
Normal weight	2785	(74.9)	1159	(75.7)	1301	(73.5)	581	(72.8)	1481	(76.2)	578	(78.9)
Overweight and obesity	599	(16.1)	226	(14.8)	289	(16.3)	130	(16.3)	310	(15.9)	96	(13.1)

*mean* Mean value, *SD* Standard deviation, age in years, *IOTF* International Obesity Task Force

**Fig. 3:**
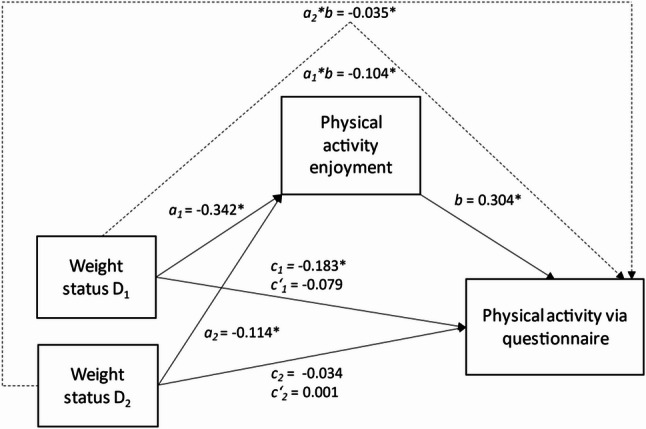
Path diagram for mediation model with questionnaire-based data, adjusted for age, sex and socioeconomic status. Legend: Standardized coefficients are presented. D1 = overweight vs. normal weight, D2 = underweight vs. normal weight, c_1_ and c_2_ = total effect of weight status on physical activity, a_1_ and a_2_ = effect of weight status on physical activity enjoyment, b = effect of physical activity enjoyment on physical activity, c’_1_ and c’_2_ = direct effect of weight status on physical activity while controlling for physical activity enjoyment, a_1_*b and a_2_*b = indirect effect on physical activity, * = *p* < .05

Results of the mediation analysis examining the total, direct, and indirect effects of weight status on PA regarding questionnaire-based data are shown in Fig. 3; Table 2. For path *a*_*1*_ *and a*_*2*_, the association between weight status and PA enjoyment was significant for both children with overweight and underweight. Path *b* showed a significant association between PA enjoyment and PA. For children with overweight, we observed a full mediation effect, with a nonsignificant direct effect (path *c*′_*1*_, ꞵ = − 0.079), and a significant indirect effect: *a*_*1*_*b* = − 0.104, 95% CI[–0.135, − 0.075]. For children with underweight, we found a fully mediated effect, with a significant indirect effect: *a*_*2*_*b* = − 0.035, 95% CI[–0.068, − 0.003], and a nonsignificant direct effect (path *c*′_*2*_, β = 0.001).

**Table 2:** Mediation analysis for the dimension physical activity enjoyment for questionnaire-based and accelerometer-based data

	Regression coefficients					Model summary					
Path	B	SE	β	t	*p*	R	R2	F	df1	df2	*p*
*Questionnaire-based data*											
Total effect						0.249	0.062	40.953	6	3711	<0.001
c_1_ (X→Y)	-0.324	0.078	-0.183	-4.430	<0.001						
c_2_ (X→Y)	-0.059	0.099	-0.034	-0.598	0.550						
Path a						0.296	0.087	59.221	6	3711	<0.001
Intercept	4.933	0.039		127.580	<0.001						
a_1_ (X→M)	-0.260	0.033	-0.342	-7.895	<0.001						
a_2_ (X→M)	-0.087	0.042	-0.114	-2.054	0.040						
Path b						0.383	0.147	90.954	7	3710	<0.001
Intercept	2.083	0.202		10.326	<0.001						
b (M→Y)	0.707	0.037	0.304	19.151	<0.001						
Direct effect											
c’_1_ (X→Y)	-0.140	0.075	-0.079	-1.872	0.061						
c’_2_ (X→Y)	0.002	0.095	0.001	0.019	0.985						
Indirect effect	**B**	**SE**	**β**	**LLCI**	**ULCI**						
a_1_*b (X→M→Y)	-0.184	0.027	-0.104	-0.135	-0.075						
a_2_*b (X→M→Y)	-0.061	0.030	-0.035	-0.068	-0.003						
*Accelerometer-based data*											
Total effect						0.229	0.053	14.099	6	1524	<0.001
Intercept	89.130	2.127		41.913	<0.001						
c_1_ (X→Y)	-3.910	1.785	-0.156	-2.191	0.029						
c_2_ (X→Y)	-6.082	2.150	-0.243	-2.829	0.005						
Path a						0.300	0.090	25.143	6	1524	<0.001
Intercept	4.974	0.063		79.473	<0.001						
a_1_ (X→M)	0.258	0.053	-0.343	-4.908	<0.001						
a_2_ (X→M)	-0.097	0.063	-0.129	-1.533	0.125						
Path b						0.292	0.085	20.301	7	1523	<0.001
Intercept	57.706	4.741		12.173	<0.001						
b (M→Y)	6.318	0.856	0.190	7.385	<0.001						
Direct effect											
c’_1_ (X→Y)	-2.281	1.768	-0.091	-1.290	0.197						
c’_2_ (X→Y)	-5.469	2.115	-0.218	-2.586	0.010						
Indirect effect	**B**	**SE**	**β**	**LLCI**	**ULCI**						
a_1_*b (X→M→Y)	-1.629	0.436	-0.065	-0.100	-0.034						
a_2_*b (X→M→Y)	-0.613	0.375	-0.025	-0.055	0.003						

*X* Weight status, _*1*_ Overweight vs. normal weight, _*2*_ Underweight vs. normal weight, *M* Physical activity enjoyment, *Y* Physical activity, *B* Unstandardized regression coefficient, *SE* Standard error, *β* Standardized coefficient, *df1* Degrees of freedom of the numerator, *df2* Degrees of freedom of the denominator, *LLCI* Lower limit of the 95% confidence interval, *ULCI* Upper limit of the 95% confidence interval. Due to dichotomous predictors, β for indirect effects are represented as partially standardized relative indirect effects

**Fig. 4:**
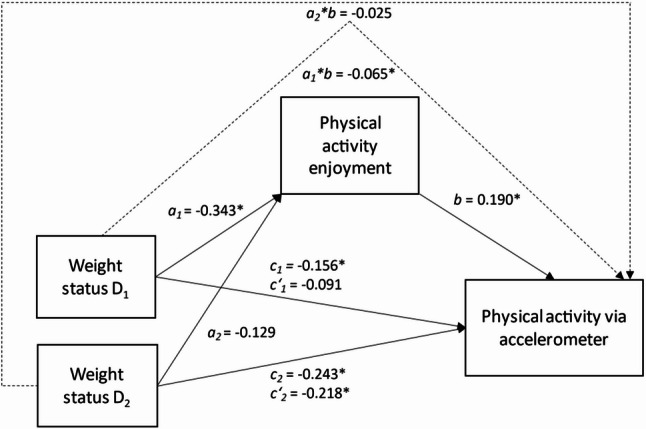
Path diagram for mediation model with accelerometer-based data, adjusted for age, sex and socioeconomic status. Legend: Standardized coefficients are presented. D1 = overweight vs. normal weight, D2 = underweight vs. normal weight, c_1_ and c_2_ = total effect of weight status on physical activity, a_1_ and a_2_ = effect of weight status on physical activity enjoyment, b = effect of physical activity enjoyment on physical activity, c’_1_ and c’_2_ = direct effect of weight status on physical activity while controlling for physical activity enjoyment, a_1_*b and a_2_*b = indirect effect on physical activity, * = *p* < .05

Regarding accelerometer-based data, the association between weight status and PA enjoyment was significant only for children who were overweight (*a*_*1*_), but not for those with underweight (*a*_*2*_). Path *b* showed a significant association between PA enjoyment and PA. In children with overweight, we found that the association between weight status and PA is fully mediated by PA enjoyment, as indicated by a nonsignificant direct effect (path *c*′_*1*_, β = − 0.091) and a significant indirect effect: *a*_*1*_*b* = − 0.065, 95% CI[–0.100, − 0.034]. For children with underweight, the indirect effect was not statistically significant (*a*_*2*_*b* = − 0.025, 95% CI[–0.055, 0.003]), but the direct effect was significant (path *c*′_*2*_, β = − 0.218) (Fig. 4).

## Discussion

Our analysis revealed that PA enjoyment mediated the association between weight status and PA: Children with overweight reported lower PA enjoyment compared to children with normal weight, and lower PA enjoyment was in turn linked to lower PA. This confirmed our hypothesis in both questionnaire-based and accelerometer-based measures. For children with underweight, however, the evidence was inconsistent, with mediation emerging only in the questionnaire-based data. This suggests that PA enjoyment may play a particularly relevant role in understanding lower PA levels among children with overweight, whereas its role in children with underweight remains less clear.

Results regarding children with overweight align with Leone and Ward [44], who showed that lower enjoyment mediated the link between obesity and PA, though their study focused only on adult women and exercise enjoyment, which differs from our broader measure of PA enjoyment. According to the concept analysis by Bajamal and colleagues, PA enjoyment depends on biological, psychological, and behavioural antecedents – conditions that must be present for PA enjoyment to emerge in the first place [45]. For children with overweight or obesity, several of these antecedents may be compromised. For example, previous research has shown that children with overweight or obesity often experience lower self-esteem, higher levels of anxiety and stress, or have a negative body image perception [8], and individual factors identified as relevant antecedents to PA enjoyment [45]. These conditions may inhibit the development of positively valanced emotions towards PA. Thus, weight status may reduce PA enjoyment indirectly by compromising these antecedents, which in turn reduces PA, consistent with our results. Future work should therefore focus on developing and testing theoretical frameworks that explicitly incorporate antecedents of PA enjoyment, in order to better understand how overweight in children may compromise these conditions and thereby reduce PA enjoyment and subsequent PA behaviour.

Whereas the role of PA enjoyment in children with overweight seems more consistent, findings for underweight are less clear. One study from Malaysia reported higher PA enjoyment among children with underweight compared to their normal-weight peers [20]. However, given differences in study design, measurement, and context, as well as limited theoretical grounding, the comparability and generalizability of these findings are restricted. In contrast, our results indicate lower PA enjoyment among children with underweight, which is consistent with findings that this group may show lower motivation to exercise and more negative weight perceptions [46].

Furthermore, evidence for the association between underweight and PA is inconsistent and differs by sex, age, and PA domain [9–11, 47]. In high-income countries, underweight is often found in children from higher SES families [48], possibly because these families are more health aware and encourage PA [49]. This may result in PA levels comparable to those of children with normal weight, or even excessive PA levels if body image concerns or mental health conditions related to body weight are present [50]. Such heterogeneity may explain why PA enjoyment did not consistently mediate the association in our sample.

While in our study, children with underweight showed less PA; this was only significant for the total path in the accelerometer-based data. The discrepancy of results between questionnaire- and accelerometer-based data may be due to methodological differences. Accelerometer data were continuous, whereas questionnaire categories used cut-offs, potentially reducing sensitivity to detect group differences [51]. Smaller subgroup sizes, especially for underweight, may also have limited statistical power. Further research is needed to understand the mechanisms and potential relevance of underweight in relation to PA in children.

Independent of weight status category, the standardized beta values in our models were relatively small [52], which raises questions about the strength and practical relevance of the observed effects. Here, the dual-mode theory by Ekkekakis may provide a useful approach. According to this theory, feelings towards PA change depending on the intensity [53]. In one study, PA enjoyment during high-intensity exercise was compared to that during moderate-intensity exercise, with high-intensity interval training being rated as substantially less enjoyable by women with overweight [54].

Understanding at which intensities and in which contexts PA enjoyment in children with overweight occurs could help tailor interventions more effectively. This includes exploring whether PA enjoyment varies across activity types, such as structured sports versus free play, and whether access to preferred activities influences this association. The stronger effects observed for children with overweight compared to those with underweight may be explained by social and behavioural mechanisms. Children with overweight are more likely to encounter stigmatization, negative feedback, or exclusion in the context of PA [8], which can diminish their PA enjoyment and thereby amplify the mediating role of PA enjoyment. Moreover, overweight is generally associated with more pronounced reductions in PA levels than underweight [47], giving PA enjoyment greater potential to explain group differences. Together, these mechanisms may account for the stronger and more consistent mediation effects found in children with overweight.

Taken together, these insights underline the need for prevention strategies that not only promote PA but also explicitly foster PA enjoyment, which can be enhanced through perceived competence, perceived social interaction, novelty experience, and perceived physical exertion [55, 56]. While social and motivational barriers frequently hinder PA engagement in children with overweight [8], it is important to also consider their existing strengths. Activities that emphasize absolute muscular strength, an area where children with overweight tend to perform better than their normal weight peers, may lower barriers and enhance PA enjoyment [57]. Furthermore, new approaches such as exergaming appear to be a strategy for providing accessible sports activities for children with overweight and encouraging them to take part in PA [58].

The strength of our study lies in its large sample size of a nationwide cohort. Furthermore, objectively measured weight and height by trained staff increase the validity of BMI measurements and minimizes the risk of bias. For our outcome, we used self-reported questionnaire-based data as well as device-based data via an accelerometer. This combination captured different constructs of PA: self-reported data reflect perceived PA patterns, while accelerometer-based data provide device-based records. Their complementary use offers more comprehensive insights into children’s movement behaviour. Lastly, we pre-registered the hypotheses and the analysis plan to limit bias. Nonetheless, some limitations must be considered when interpreting the results. While the study followed a standardized measurement procedure, we did not account for the weight of clothing, which could lead to a slight overestimation of BMI values [59]. The use of BMI data instead of bioimpedance analysis may under- or overestimate prevalence in weight status, which could lead to different results in our analysis [60]. Due to the cross-sectional study design, reverse causation is possible. Furthermore, causality cannot be assumed, which is why the results of the mediation analysis in particular must be interpreted with caution [43]. The MoMo 2.0-Study’s primary focus on PA and physical fitness may have led to selection bias, as children who were less interested in these topics might have been less likely to participate.

## Conclusion

To summarize, our findings suggest that diminished PA enjoyment partly explains why children with overweight are less active, underlining the importance of considering motivational factors in this pathway. Longitudinal studies are required to clarify the causal relationship of the associations between weight status, PA enjoyment, and PA itself. By examining the potential role of PA enjoyment in the association between weight status and PA in children, this study adds evidence that can guide intervention strategies and the design of preventive programs. Tailored public health messaging should address reduced PA enjoyment in children with overweight by framing PA around fun, mastery, and positive experiences, thereby reducing motivational disparities and supporting long-term effectiveness of interventions.

## Data Availability

The dataset is subject to access restrictions to ensure data protection and compliance with ethical standards. Access can be granted upon a justified request and after signing a data use agreement. Requests should be addressed to the principal investigator of the MoMo 2.0-Study. Each request will be reviewed individually to ensure that the proposed use is consistent with the study’s objectives and ethical guidelines. The codebook is not publicly available, as it is continuously updated to reflect ongoing data integration and variable refinement. To ensure access to the most recent and relevant version, we kindly ask interested researchers to contact the data management team of the MoMo 2.0-Study. We are happy to provide tailored documentation and guidance on the most relevant variable sets based on the intended research use. Statistical analyses were performed using R (version 4.4.2) and RStudio (version 5.1.513) (R Core Team, 2021; RStudio Team, 2021). The code used to generate the study results is available upon reasonable request from the corresponding author. Please note that access may be subject to institutional or data-sharing agreements.

## Abbreviations

BMI: Body Mass Index
IOTF: International Obesity Task Force
MoMo-PAQ: MoMo Physical Activity Questionnaire
MVPA: Moderate to vigorous physical activity
PA: Physical activity
PACES-S: Short version of the Physical Activity Enjoyment Scale
SES: Socioeconomic status
